# Chicago Medical Society

**Published:** 1882-09

**Authors:** 


### Society Reports.

### ARTICLE IV.

CHICAGO MEDICAL SOCIETY.

Stated Meeting, held August 7, 1882. Dr. G. C. Paoli, President *pro tem.*

### TWO CASES OF CIRRHOSIS OF THE PANCREAS IN PRIVATE PRACTICE, BY DR. C. W. EARLE.

Case I.—Mr. G. E., æt. sixty-five, in November, 1880; temperate; no history of syphilis. Began to emaciate at beginning of 1880; did not care to eat meat; was weak, short of breath; tongue white; and there was a murmur with the first sound of the heart. Slight pain manifested on pressure at the epigastrium. Was occasionally sick at the stomach. Legs slightly œdematous. Stools black; occasionally containing little lumps of blood and mucus. No diagnosis possible from the symptoms present. Treatment consisted of tonics, mineral acids, and a generous diet. Dr. N. S. Davis saw the case in consultation. Both came to the conclusion that there was some grave affection of the digestive system which would probably result in death. Patient died in the course of a month.

*Post-mortem.*—Marked whiteness of all the tissues. Pancreas white and indurated. It was examined by Dr. S. J. Holmes, who found it contracted; abnormal in consistency; fibrous to touch. The microscope showed much increase of connective tissue, with obliteration of certain glandular vesicles and atrophy of others, resulting from the compression exerted by the newly formed connective tissue. There was slight, fatty degeneration of the parenchyma of the kidney.

Case II.—Mr. S. K. R., æt fifty-seven. Two years ago began to fail in strength, but continued business till within two months of his death. Had had hæmorrhoids for five or six years. Had been troubled with indigestion for some years, and had a bilious attack a year ago. In February, 1882, he passed considerable blood by the rectum, and continued passing blood in his stools, and suffered much pain at the same time. Lost sixty pounds; skin white; pulse, respiration and temperature normal. Antipathy to meats; gained a few pounds under treatment with tonics, mineral acids, pancreatine and pepsin. He had alternate diarrhœa and constipation; pulse was as low as 55 at times. Later he was treated by massage, with relief of his bowel affection. Pain remained near the ensiform cartilage. He now expectorated a large amount of mucus, and vomited a large amount of coagulated blood; probably two pounds. He died June 30.

*Post-mortem.*—The abdomen alone was opened. There was considerable fat between the integument and abdominal muscles; whiteness of tissues. The pancreas was hard, enlarged, and filled with spots, plainly seen with the naked eye. The bowel was somewhat contracted in some places; no trace of disease of the rectum. The hæmatemesis had probably been caused by pressure of the head of the pancreas upon some blood-vessel, but there was no breach of continuity in the mucous membrane of the stomach.

The pancreas measured nine inches in length, was denser and larger than normal, and presented numerous yellow-white spots one-fourth or one-half inch in diameter. Marked increase of the pancreatic connective tissue.

Dr. L. C. Waters reported a complicated case of scarlatina, which came under his treatment February 23, 1882. The patient, æt. eight years and three months, had a chill, a soreness of the throat, a temperature of 104 2-5, and a pulse of 126. There was constipation and vomiting. The tonsils were swollen. A scarlatinous eruption appeared the next day, when the temperature arose to 105 9-10, and there was delirium. In the course of eight days, under ordinary treatment, the patient was better, and the eruption had disappeared. On ninth day, patient fell worse; his pulse beat 150 a minute; the temperature was 102; there was fœtor of the breath, and there was considerable swelling of the throat externally, extending to the cheeks on both sides. A false membrane formed in the throat. Upon removing a portion of this, there was bleeding from the exposed surface. Twenty-eight hours later the patient was dead, no treatment being of any avail.

The treatment adopted in this case was as follows: At first a cathartic was given for the constipation. Patient was sponged thoroughly all over the body with tepid water three times a day. Cold applications were made to the head. Quininæ sulphas in doses of two grains every four hours; tinct. of iron and potassii chloras were also given. Cold applications were made to the throat. The doctor regarded the last complication as diphtheria, which was sometimes liable to arise in cases of scarlatina.

Dr. R. N. Isham reported a case of rupture of the left kidney, with autopsy, and the society adjourned for the rest of the summer.

